# A multidisciplinary protocol for reducing excessive and maintaining a healthy body weight in the personalized management of chronic diseases in children and adults

**DOI:** 10.1371/journal.pone.0306400

**Published:** 2025-03-13

**Authors:** Ivana Banić, Marija Jankić, Adrijana Miletić Gospić, Katarina Pentek, Petra Anić, Tajana Burkuš, Krešimir Hrg, Karmen Zadro, Jelena Miličević, Ivana Šuljić, Marcel Lipej, Davor Plavec, Lenkica Penava, Mirjana Turkalj

**Affiliations:** 1 Department of Translational medicine, Srebrnjak Children`s Hospital, Zagreb, Croatia; 2 Department of Nutraceuticals, Belupo Pharmaceuticals and Cosmetics Inc., Koprivnica, Croatia; 3 Department of Nutrition, Srebrnjak Children’s Hospital, Zagreb, Croatia; 4 Culinary Department, Podravka Food Industry Inc., Koprivnica, Croatia; 5 Research Department, Srebrnjak Children’s Hospital, Zagreb, Croatia; 6 Faculty of Kinesiology, University of Zagreb, Zagreb, Croatia; 7 IT Department, Srebrnjak Children’s Hospital, Zagreb, Croatia; 8 Faculty of Medicine, J.J. Strossmayer University of Osijek, Osijek, Croatia; 9 Catholic University of Croatia, Zagreb, Croatia; 10 Department of Pediatric Allergy and Pulmonology, Srebrnjak Children’s Hospital, Zagreb, Croatia; University of Montenegro: Univerzitet Crne Gore, MONTENEGRO

## Abstract

Despite regularly used treatment asthma in both children and adults is not fully controlled. This is more prominent in certain disease subtypes, such as obese asthma. The vast complexity of asthma phenotypes and often overlapping endotypes emphasizes the need for implementing the concept of precision medicine in disease management. In order to address these concerns, an innovative and personalized programme for reducing excessive and maintaining a healthy body weight will be developed by experts from pharmaceutical and food industry (Belupo Inc. and Podravka Inc., Croatia) as well as clinical experts (Srebrnjak Children`s Hospital, Croatia). The programme will involve meals replacement (standard and innovative formulas), dietary program and nutritional counseling, physical activity and other individually tailored measures according specific disease phenotypes and underlying pathophysiological mechanisms in each participant. The outcomes of this study should contribute to better control of the underlying condition, reduction of the frequency and severity of symptoms and, consequently, in the improvement of the participant’s overall quality of life.

## Introduction

Asthma is the most common chronic disease in children, and numerous epidemiological studies show a constant increase in the incidence of asthma, with an almost certain continuation of a similar trend in the future [1]. Due to the complex and heterogeneous nature of the disease, there is still no cure and treatment is primarily symptomatic, serving to alleviate current symptoms, control inflammatory processes underlying the disease and prevent acute exacerbations. Currently, treatment outcomes in asthma exhibit a high degree of variability and depend largely on the individual characteristics of the patients [2]. Despite numerous studies on certain subtypes of asthma, knowledge about the causative agent and etiological factors is still insufficient, and therefore it is impossible to make a decision on the treatment regime that would suit an individual the most and to achieve optimal asthma control [2]. One of the most common comorbidities in asthma is obesity. In the last few decades, there has been an increase in the number of obese people to the extent of a global epidemic. This has also been observed in children, and the increasing trend continues as it has tripled over the last thirty years. Research has shown that excess body weight is an independent factor in the development of asthma and that there is a cause-and-effect relationship between body mass index and asthma. More than 40% of asthmatics are obese, and excessive body weight is a risk factor that leads to more frequent and severe symptoms [3,4]. Epigenetic modifications are considered one of the key triggers of pathophysiological mechanisms in asthma. It is believed that epigenetic modifications act as mediators of one’s exposure to environmental factors such as unhealthy diet and lifestyle that lead to excessive weight and obesity. Other than being a secondary mechanism (consequence) caused by disease pathophysiology, epigenetic mechanisms (such as DNA methylation and small non-coding RNA expression) may act as predisposing factors for asthma, and more specifically, asthma-related phenotypes (and specific endotypes) such as “obese asthma” [5]. Obesity is also associated with increased inflammation of the adipose tissue that may lead to an increase in systemic inflammation, with a number of immune cells and inflammatory mediators involved in this immune dysregulation [6].Current nutritional guidelines for weight reduction and maintenance of normal weight are based upon changes in lifestyle- energy-deficient diet, and regular physical activity. In situations where it is impossible to achieve a reduction in body mass by applying these guidelines, products with a reduced energy value, from the food category for special nutritional needs, can be used [7]

The aim of this research is to design and develop a personalized concept (regime) for weight reduction and maintaining a healthy body weight in order to gain better insight into the heterogeneity of disease subtypes under the broad term of “asthma”. More specifically, this research aims to identify new molecular and pathophysiological mechanisms involved in the development of asthma and its specific phenotypes which could, in turn, enable more effective management of the disease. Additionally, this research will potentially contribute to earlier and more precise diagnostics, optimization of current and development of novel, personalized treatment options in patients with specific asthma phenotypes (“obese asthma”). We hypothesize that weight reduction will help achieve better disease control and better treatment outcomes in both children and adults with obese asthma.

### Study endpoints

The primary endpoint of this study is to determine the effectiveness of a weight reduction diet program based on an innovative and standard product line (meal replacements) through actual reduction of the body mass (BM)/ fat mass (FM) of the participants in relation to their pre-trial body mass (BM)/fat mass (FM).

The secondary study endpoints include:

1. reduction of waistline and reduction of waist/hip and waist/height ratio;
2. improvement of the participants` lipid profile (increase in HDL/LDL ratio) compared to initial values;
3. improvement of the quality of life of participants;
4. improvement of asthma control, decrease in the number and severity of asthma exacerbations in participants with asthma;
5. decrease in systemic inflammation, decrease in oxidant stress biomarkers.

## Materials and methods

Research and development activities within this study will take place at the Srebrnjak Children`s Hospital, Zagreb, Croatia (SCH) and Belupo Inc. and Podravka Inc., Koprivnica, Croatia over 33 months and encompass four different phases: (I) development of a formulation for food products intended for the loss of excess body weight, (II) development of a personalized program for excess body weight, (III) clinical study, and (IV) development of multidisciplinary nutritional support.

The first research phase employs the defining of the target product composition (food products for body weight reduction). In the following phase, based on the established functional-technological characteristics of the products, a long-term programme for excess body weight reduction will be developed. The program will be adaptable to the personalized needs of different target groups of participants.

In the third phase of research activities a clinical study will be conducted. In the final phase of this research a personalized multidisciplinary support programme (with a counseling center) for the reduction of excessive body weight will be created encompassing innovative food products for weight loss.

### Study population

Overweight and obese participants (adults and children) will be recruited in a prospective interventional case-control clinical study at the Outpatient clinical at Srebrnjak Children`s Hospital, Zagreb, Croatia. The sample size is set to 240 participants, adults (>18 years) and children aged 12-18 years, with 1/3 of the participants being children (approximately 160 adults and 80 children). Written informed consent will be obtained from all participants and their parents/caregivers. The study was approved by the local Ethics committee at SCH (on 14^th^ March 2022, Ref. no.: 04-301/1-22; Supplementary material). The study has been registered at ClinicalTrials.gov PRS (ID: NCT05980663, NCT05733871).

The study involves participants (adults and children aged 12 to 18 years, of both sexes) who have themselves signed a written informed consent (for pediatric participants both the children and their parents/caregivers will sign the informed consent, according to local legislation), i.e., those who agreed to participate in the study and do meet the criteria for inclusion. The participants will be assessed for inclusion and exclusion criteria on their first study visit after signing the informed consent. Criteria for inclusion and exclusion will be assessed by a specialist physician (pediatric allergy specialist) and an occupational medicine specialist.

The main inclusion criteria are overweightness and obesity (for adults- BMI > 27 kg/m^2^, for children- ≥ 90^th^ BMI percentile), to be able to correct for differences in body composition (e.g., higher BMI due to higher muscle mass in semi-professional or professional athletes). Additional inclusion criteria will include: physician diagnosed asthma (according to ERS/ATS guidelines) for at least one year, being on a stable dose of anti-inflammatory treatment for at least one month according to GINA (Global Initiative for Asthma) guidelines [8,9], clinically significant allergy to indoor and outdoor allergens, with positive skin prick test (SPT) and specific IgE levels (>0.7 kUA/L).

Exclusion criteria for adults are: acute respiratory infection; use of systemic corticosteroids, antidepressants, cytostatics, hormone (replacement) treatment, beta-blockers; recent asthma-related visit to emergency department (in the past three weeks); pregnancy and breastfeeding; coexistence of other serious chronic illnesses, such as uncontrolled diabetes mellitus requiring insulin therapy and other endocrine disorders, cardiovascular disorders and other chronic diseases (including malignancies), chronic inflammatory diseases of the gastrointestinal tract, mental disorders; currently on or recently gone through a weight loss programme (in the past 3 months); an unwanted or uncontrolled loss of body weight > 5% in the past 6 months; bariatric surgery in the past 6 months; use of medications that influence appetite; eating disorders; unstable thyroid disease.

Exclusion criteria for children are: known inborn or perinatal pulmonary disease; known congenital and other serious chronic illnesses (such as malignant diseases, chromosomal aberrations, neurological disorders that affect oral motor skills, chronic gastrointestinal disorders or severe metabolic disorders), pulmonary malformation; oxygen therapy after birth with a duration of more than 24 h; ventilator support or mechanical ventilation after birth; diagnosis of cystic fibrosis; primary ciliary dyskinesia; heart failure diagnosed after birth affecting pulmonary circulation; major respiratory diseases such as, e.g., interstitial lung disease. Moreover, participants were excluded from study visits and biomaterial collection in the case of fever of at least 38.5 °C during the last two weeks prior to the planned visit.

### Assessments, measurements, diagnostic procedures and data collection

Recruitment started on 17^th^ January 2023. Upon recruitment, after the participants themselves and their parents/caregivers have agreed to participate in the study and signed the informed consent form, participants will undergo a physical examination, anthropometric measurements (height and weight for calculation of BMI and BMI percentiles, hip and waist circumference measurement, body composition assessments by Tanita MC-780MA multi frequency segmental body composition monitor based on bioelectrical impedance analysis (BIA) and by 7-site skinfold measurement using Harpenden skinfold caliper) along with other routine diagnostic tests as part of their asthma management plan, such as allergy tests (skin prick test and total and allergen-specific immunoglobulin E in the serum), lung function and bronchial challenge tests (spiroergometry) etc. Additionally, whole blood samples will be taken for subsequent transcriptomic analysis and isolation of PBMCs (peripheral blood mononuclear cells), as well as stool samples for metagenomic analysis of the gut microbiome. Blood samples leftover after routine diagnostic tests (blood count, lipid profile, glucose level etc. (Table 1) will be stored at -20°C for subsequent genetic analysis or at -80°C for evaluation of systemic inflammation. Stool samples will be stored at -20°C for subsequent microbiome analysis. Other relevant clinical data and data such as personal and family history, and demographics will also be collected.

**Table 1:** Features used in this study.

Baseline demographics	Gender, age
**Subjective clinical data**	Personal and family medical history, atopy status, allergic rhinitis (AR), atopic dermatitis (AD), food allergy and other comorbidities
**Objective clinical data**	Skin prick and total and specific e.g. test results, hematologic and biochemical blood test results (blood count, lipidogram, glucose level, creatinine, urates, total bilirubin, ALT^1^, AST^1^, GGT^1^, ALP^1^, electrolytes), urine analytes (with ketone bodies), comorbidity (ENT^2^ examination, pH probing with impedance for the reflux episodes monitoring for diagnostics of GERD/LPR^3^, other) lung function testing, FENO^4^, bronchial challenge tests, spiroergometry, treatment used, nutritional status (vitamins, minerals)
**Anthropometric data**	Height, weight, BMI^5^, BMI percentile for children, hip and waist circumference and hip/waist ratio, body composition (% fat mass, % fat free mass)
**Systemic inflammation data**	hsCRP^6^, leptin, other serum markers
**Genetic data**	Epigenetic status (DNA^7^ methylation), genetic expression patterns, gut microbiome
**Dietary data**	Dietary intake, food diaries, dietary questionnaires

^1^Liver enzymes (Alanine Aminotransferase, Aspartate transaminase, Gamma-glutamyl transferase, Alkaline phosphatase).

^2^Ear, Nose, and Throat examination.

^3^Gastroesophageal reflux disease/laryngopharyngeal reflux.

^4^Fraction of Exhaled Nitric Oxide.

^5^Body Mass Index.

^6^C-reactive protein.

^7^deoxyribonucleic acid.

Their dietary patterns and food intake will be assessed by food frequency questionnaire (FFQ), 14-point Mediterranean Diet Adherence Screener- MEDAS (or children version – KIDMED), The Three-Factor Eating Questionnaire (TFEQ-R1) and 3-day food diary at the beginning and end of the study.

Participants will be assigned specific and adapted physical exercise programmes and vital and other parameters (heart rate, respiration, step count etc.) will be collected by smart bands.

### Follow up

Participants will be followed up at regular intervals. Every two months they will undergo a medical physical examination and anthropometric measurements once a month (as discussed in the previous section) and any adverse events recorded on adverse events reporting forms will be discussed. Additionally, their dietary intake and study compliance will be assessed by 3-day food diary and 24-hour dietary recall in each phase of the study. For better adherence to the programme, individual nutritional consultations will be carried out if needed.

**Fig 1:**
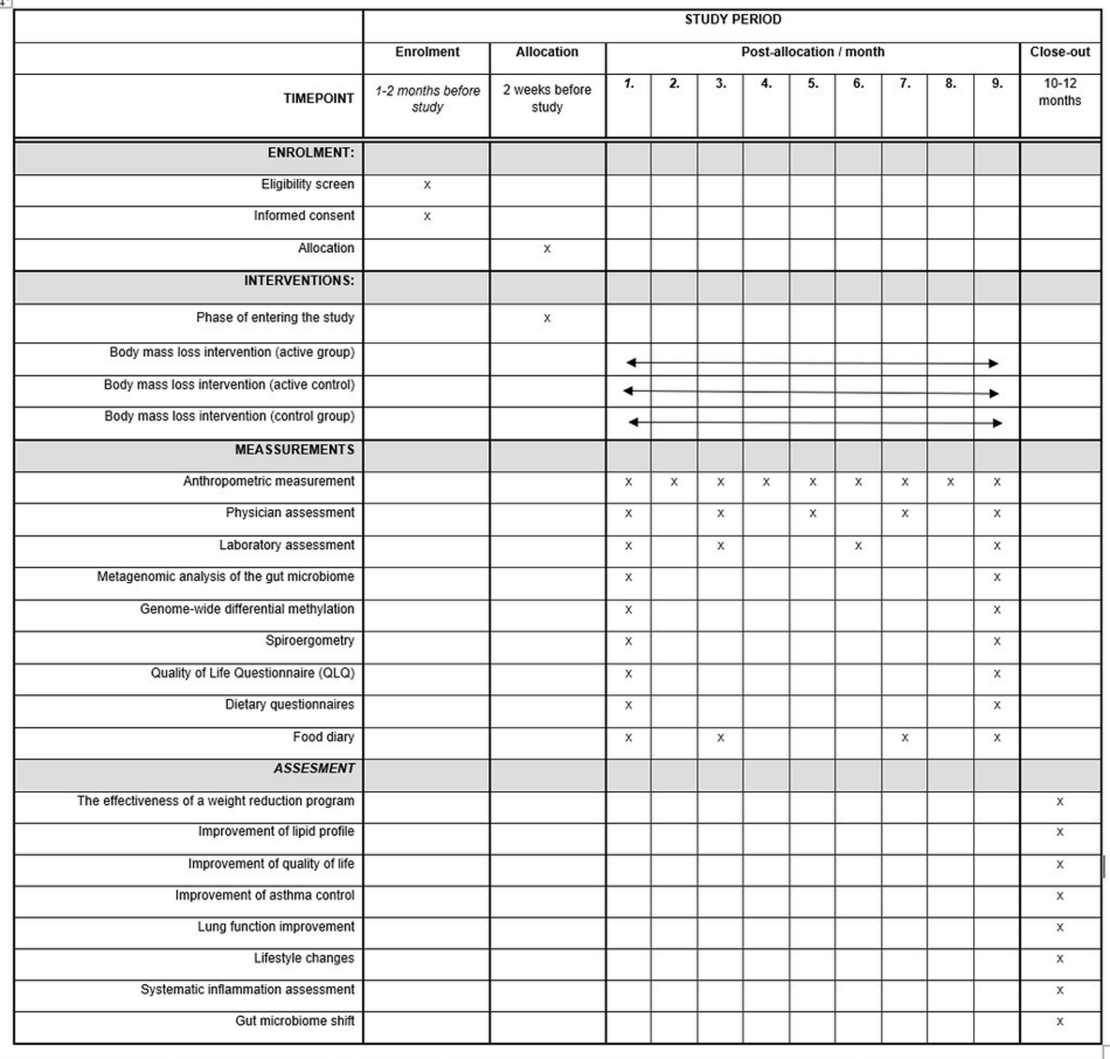
Study design and key timepoints.

After 1, 3, 6, 9 months and at the end of the study, the participants will have blood and urine samples collected. At these timepoints, the participants health status, nutritional status and level of systemic inflammations will be assessed. Stool samples will be taken at baseline and at the end of the study. Design and key timepoints of the study are presented in Fig 1.

The appearance of any exclusion criteria during the follow up phase will be a signal for participant exclusion from the study. During the entire course of the study, it will not be allowed to use other weight loss methods such as weight-loss medications or bariatric surgery.

Upon recruitment, participants will be randomized into 3 groups, according to weight loss plan: innovative formulation (1); standard (2) and control group (3) in a ratio of 2:2:1, respectively. Stratified randomization according to age, sex and baseline BMI will be provided to secure balanced groups according to their characteristics at baseline. Randomization plan will be done by the independent statistician not involved in recruitment and participants assessment prior to any recruitment. Trial participants, principal researchers, outcome assessors and data analysts will be blinded after intervention group allocation. Unblinding is permissible in case of severe adverse events or study withdrawals.

**Innovative group (1):** participants will use the innovative line of products intended for body weight reduction, that represent a meal replacement for weight management (liquid formulations and powders for resuspension- shakes and soups)- test group;
**Standard group (2):** participants will use the standard line of products intended for weight reduction that represent a meal replacement for weight management with already proven clinical effectiveness (positive control);
**Control group (3):** participants will receive personalized programme for a reduced low-calorie diet (balanced nutrition).

The standard product line will contain common ingredients and is currently commercially available [10], while the innovative product line differs will implement raw materials that are considered better sources of potentially bioactive components. Clinical studies suggest that these ingredients could have more health benefits due to their anti-inflammatory and antioxidant properties, including an impact on clinical parameters of the metabolic syndrome that is increasingly present in obese individuals [11].

All participants will implement physical activity regimes, personalized according to age, gender, health status, BMI, body composition, current physical fitness etc.

**Fig 2:**
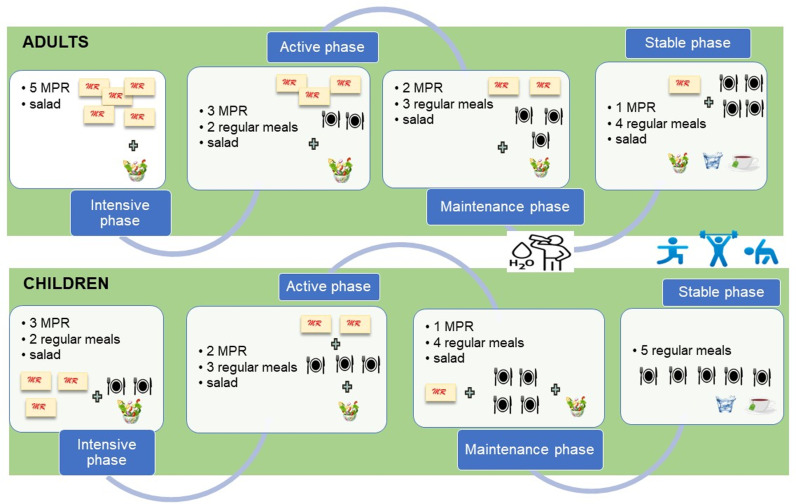
Study phase diagram for active group (innovative and standard). Control group (not shown) has 5 regular low-calory balanced meals throughout the entire course of the study. In each phase. exercise and sufficient fluid intake are required.

The study protocol is defined in detail in Supplementary material. This study will encompass 4 phases of different duration and design for adults and pediatric participants): initial phase (intensive), active phase, stable phase and monitoring phase, preceded by an entry phase of adaptation to the study protocol (duration of entry phase: 2 weeks). The study phase diagram is shown in Fig 2.

### Statistical analysis

Sample size was calculated as 88: 88: 44 (N = 220 with the test for repeated measures using effect size of D = 0.5 (which represents a medium size clinical effect), SD = 1, power = 0.8 and alpha = 0.05 (minimum group size of 34) using a 15% drop-out rate. Analysis of variance for repeated measurements will be used to determine the effectiveness of individual programs on primary outcomes (BM/FM). The same analysis will be used to analyze secondary performance parameters and safety parameter variables. A chi-square test or Fisher’s test will be used to compare the occurrence of safety-related parameters between groups. The threshold for missing values will be set at 5%. If the missing values for certain variables exceed this threshold, these will be handled by imputing variables according to previously measured or observed values, that is the last observation will be carried forward. The analysis will be done as intention-to-treat (ITT) and as per-protocol (PP). P < 0.05 will be considered statistically significant with appropriate correction for multiple comparisons. The statistical analysis will be performed using STATISTICA 12 program (StatSoft, Tulsa, OK, USA).

## Results

The data that will be collected in this study is shown in Table 1.

At the end of the study, the results of diagnostic tests, anthropometric and other measurements will be analyzed and compared with clinical, molecular (genetic and epigenetic) data and other data collected during the study (such as data on physical activity). Additionally, all results will be integrated into an innovative (bio)informatics platform. In consultation with experts in medical, molecular-genetic, bioinformatics, and food-biotechnological specialties, the clinical significance of the obtained results and their importance for each individual patient will be defined and a personalized programme for body weight reduction will be designed.

## Discussion

Despite numerous studies on individual subtypes of asthma, knowledge about the causative agents and etiological factors of the disease is still insufficient. Generalized guidelines are used, which do not provide full insight into the specific biological processes of asthma. The financial costs of asthma treatment are significant and include direct medical (diagnosis, medications, hospitalization) and indirect costs, including reduced quality of life for both the patients and their families (especially in children with asthma) and reduced work ability. The concept of precision medicine implies that different asthma phenotypes and endotypes need to be identified in order to optimize and improve treatment [12]. With effective prevention programmes and therapeutic measures based on a personalized approach, it is possible to avoid most of these costs.

Obesity is a significant social problem that has expanded to a global scale over the last few decades. Additionally, obesity is a common comorbidity in asthma and an independent risk factor for asthma development [13].

Asthma symptoms in obese patients are more significant and frequently lead to acute exacerbation and impaired quality of life. Research conducted on 1,000 people showed that symptoms in obese asthmatics led to hospitalization five times more often than within the healthy weight asthmatics [14]. Moreover, obesity in asthma represents a special phenotype of the disease called “obese asthma”, with complex relationships between metabolic and immune dysregulation (pro-inflammatory) [15].

Despite the fact that the clinical manifestations of this phenotype are well described, the etiological and pathophysiological mechanisms underlying obese asthma remains elusive. This study will aim at identifying new pathophysiological mechanisms involved in the development of the disease and enable better monitoring of the progress, degree of asthma control and risk for exacerbations asthma, and treatment success. The purpose of this study is design and develop an improved and personalized management approach that focuses directly on the causes of the diseases and not just the symptoms. Weight loss in obese individuals with diagnosed asthma is associated with symptom improvement and reduction of medication use [16]. Evidence shows that structured, personalized body weight reduction programs that include nutritional interventions (such as meal replacements and the introduction of a balanced diet) and lifestyle changes (including physical activity) may have a significant effect on improving the health status of the subjects as well as reducing the risk of cardiovascular diseases and metabolic syndrome [17].

The outcomes of this study may significantly contribute to the improvement of health care utilization, reducing the financial burden for the cost of treatment due to the decrease in the number of chronic patients with pronounced symptoms and the number of cases requiring hospitalization and ultimately improve the quality of life of vulnerable groups (children, patients). Furthermore, the results of this study will provide better insight into the regulatory mechanisms driving inflammation in obesity and asthma and may will serve as a basis for further research on the same or related topic, as well as research into environmental and intrinsic factors with the development of asthma in children and their association with global and gene-specific epigenetic variations.

All study results and newly acquired knowledge will lead to the development of multidisciplinary nutritional support in the form of a counseling center that will help improve nutritional and other guidelines that will be individually tailored according to specific needs of each patient and disease subtype. One of the possible limitations of our study may be a significant drop-out rate. As the study protocol is intense at certain phases, a certain proportion of the participants may lose interest in participating in the study and not show up for regular check-up visits. This will be mitigated by recruiting a larger number of participants in the clinical study in both child and adult groups at baseline to account for drop-outs. Another way of minimizing the drop-out rate will involve continuous psychological support provided by experts in the field of eating behaviors and cognitive behavioral therapy. additionally, since the first phase of the study is the most intensive one (stringent dietary intake) and has the most meal replacement products (MRP) included, it will be the hardest phase to pass. Therefore, the MRPs will be designed to be of appealing taste and texture.

An additional bias might be over representation of the female gender in each of the populations, as asthma after puberty is more common in women (girls) than in men (boys).

## Conclusions

The implementation of the results of this study will enable timely and precise diagnosis of the asthma and its specific subtypes (obese asthma), as well as the potential recognition of predispositions for their development.

## Supporting information

S1 Data Approval-Ethics committee. (DOCX)

S1 Fig A brief structured summary of the study protocol. (TIF)

S2 Table Supporting information file. (DOCX)
