# Supplementary material for: A multidisciplinary protocol for reducing excessive and maintaining a healthy body weight in the personalized management of chronic diseases in children and adults
Source: PLoS One. 2025 Mar 13;20(3):e0306400. doi: 10.1371/journal.pone.0306400 (PMC11906058; doi:10.1371/journal.pone.0306400)
Supplement: S1 Data — (DOCX) [file pone.0306400.s001.docx]

Figure S1. Approval of the Ethics Committee of the Srebrnjak Children Hospital from 14th March 2022, Reg. no.: 04-301/1-22 (in Croatian), followed by a translation of the Approval to English.

Srebrnjak Children`s Hospital

Srebrnjak 100, HR-10000 Zagreb

Croatia

Tel: +385 1 6391 100

Zagreb, 14th March 2022

Class: 100-02/22-01

Ref. No:04-301/1-22

On 14th March 2022 at the 13th Ethics Committee online session, with 5 votes PRO the members of the Ethics Committee have reached the following

**DECISION**

Article 1.

The implementation of the research project and the clinical study within the EFRR „The development of a personalized concept for the reduction of excess and maintaining a healthy body weight in the management of chronic diseases in children and adults“, Principal Investigator: Prof. Mirjana Turkalj, MD, PhD has been APPROVED hereby.

Article 2.

This decision becomes final on the day it is reached.

Elaboration

On the 9th March 2022 prof. Davor Plavec, MD, PhD has submitted the request for approval of the research project EFRR „The development of a personalized concept for the reduction of excess and maintaining a healthy body weight in the management of chronic diseases in children and adults“, funded by the European Regional and Development Fund, Competitiveness and Cohesion OP, Call for building capacities for research, development and innovation (grant agreement No: KK.01.1.1.07.0075)., along with relevant ethics related documentation. The Principal Investigator of the study is Prof. Mirjana Turkalj, MD, PhD.

According to this request, the members of the Ethics Committee of the Srebrnjak Children`s Hospital at the 13th Ethics Committee session held online on 14th March 2022 have found this study and the research project valid and ethically acceptable, thus reaching the afore mentioned Decision.

The President of the Ethics Committee of the SRebrnjak Children`s Hospital

Assoc. Prof. Prim. Helena Munivrana Škvorc, MD, PhD

____________________________________________

Deliver to:

1. The applicant

2. Archives

Table S2. Summary of the clinical study protocol in english and croatian. **Sažetak protokola kliničke studije na engleskom i hrvatskom**

| **Name**  **Naziv** | **Randomized stratified controlled clinical study for evaluating the effectiveness of a diet program designed for reducing body weight using standard and innovative products in obese participants with asthma.**  **Randomizirana stratificirana kontrolirana klinička studija procjene učinkovitosti programa prehrane namijenjenom redukciji tjelesne mase pomoću standardnih i inovativnih proizvoda kod pretilih osoba s astmom** |
| --- | --- |
| **Short name**  **Skraćeni naziv** | **Evaluation of the effectiveness of standard and innovative products that represent a substitute meal for weight management in a reduction diet** **in obese participants with asthma.**  **Procjena učinkovitosti standardnih i inovativnih proizvoda koji predstavljaju zamjenski obrok za kontrolu tjelesne mase pri redukcijskoj dijeti** **kod pretilih osoba s astmom** |
| **Study Number**  **Broj studije** | Clinicaltrials.gov No: NCT05980663, NCT05733871 |

| **Sponsor**  **Sponzor** | This study is funded by the European Regional Development Fund, the Operational Program for Competitiveness and Cohesion, calls for capacity building for research, development and innovation (KK.01.1.1.07.0075).  Projekt se financira u sredstvima Europskog fonda za regionalni razvoj, poziva Jačanje kapaciteta za istraživanje, razvoj i inovacije KK.01.1.1.07, u sklopu Operativnoga programa Konkurentnost i kohezija 2014. - 2020. (KK.01.1.1.07.0075) |
| --- | --- |
| **Principal invstigator**  **Glavni istraživač** | Prof. Mirjana Turkalj, PhD, MD– Srebrnjak Children˙s Hospital, Zagreb, Croatia  Prof.dr.sc. Mirjana Turkalj, dr.med.- Dječja bolnica Srebrnjak, Zagreb, Hrvatska |
| **Other researchers:**  **Ostali istraživači:** | Prof. Davor Plavec, PhD, MD  Željka Vlašić Lončarić, PhD, MD  Adrijana Miletić Gospić, MSc nutrition  Darija Hrnjkaš, MSc nutrition  Ivana Šuljić, MSc nutrition  Tajana Burkuš, MSc biol exp  Petra Anić, MSc biol mol  Ivana Banić, PhD, MSc in Mol Biol  Marina Jakirović, research nurse, MSc in Nurse Studies  Katarina Bogović, research nurse, MSc in Nurse studies  Lenkica Penava BSc nutrition, PhD  Katarina Pentek, BSc techn aliment  Krešimir Hrg, MSC in Kinesiology  Marija Jankić, BSc nutrition  Jelena Miličević, BSc nutrition  Karmen Zadro, BSc nutrition  Prof.dr.sc. Davor Plavec, dr.med.  Doc.dr.sc. Damir Erceg, dr.med.  Adrijana Miletić Gospić, mag.nutr.  Darija Hrnjkaš, mag.nutr.  Ivana Šuljić, mag.nutr.  Tajana Burkuš, mag.biol.exp.  Petra Anić, mag biol.mol.  Dr.sc. Ivana Banić, mag.mol.biol.  Marina Jakirović, mag.med.tech., studijska sestra  Katarina Bogović, mag.med.techn., studijska sestra  Dr.sc. Lenkica Penava dipl. ing.nutr.  Katarina Pentek, dipl.inž.preh.teh  Krešimir Hrg, mag. cin.  Marija Jankić, dipl.ing.preh.teh.  Jelena Miličević, dipl.ing.preh.teh.  Karmen Zadro, dipl.ing.preh.teh. |
| **Research institution**  **Istraživačka ustanova** | Srebrnjak Children's Hospital, Zagreb, Croatia  Dječja bolnica Srebrnjak, Zagreb, Hrvatska |
| **Supervisory Board / Ethics Committee**  **Nadzorni odbor/Etičko povjerenstvo** | Ethics Committee of the Srebrnjak Children's Hospital  Etičko povjerenstvo Dječje bolnice Srebrnjak |

**STUDY DESIGN**

**DIZAJN STUDIJE**

| **Scientific base**  **Znanstveno uporište** | In the last few decades, there has been an increase in the number of obese people to the level of a global epidemic due to inadequate diet, sedentary lifestyle, and genetic predispositions. The World Health Organization (WHO) declared obesity to be the biggest, global, a chronic health problem in adults, which is increasingly becoming a more serious problem than malnutrition (1). On the other hand, obesity is a risk factor for the development of contemporary non-infectious diseases today that account for more than 80% of mortality in developed countries, such as vascular disease, diabetes, and respiratory diseases such as asthma.  This trend is also recorded in Croatia, both in adult population and in children if the data from 2003 and 2015 are being compared (2), while according to data from 2019, almost two thirds of the adult population in Croatia were overweight or obese (42% overweight, 23% obese) (3). Of particular concern are the data from the European Obesity Monitoring Initiative from 2018/2019. according to which in Croatia 35% of children aged 8.0 to 8.9 were overweight or obese. At the national level, the problem of obesity is greater in boys than in girls, and it is 17.8% compared to 11.9% of girls. Croatia is in a high fifth place together with other countries of the Mediterranean region of Europe due to the shift from the Mediterranean to the Western lifestyle.  The modern nutritional approach to reducing excessive and maintaining normal body weight is based on changes in lifestyle, energy-deficient diet, regular physical activity, and regular control of body weight and physical activity. In cooperation with Podravka, Belupo has developed a standard and innovative product line. Products are replacing one or more daily meals, and they must comply with Regulation 2016/1413 requirements related to energy value, content, and composition of fats and proteins, as the number of vitamins and minerals in this type of product (5).  The standard product line contains common ingredients and this type of product already exists on world markets, while the innovative product line differs from the standard one by implementing raw materials that are considered better sources of potentially bioactive components. Clinical studies suggest that these ingredients due to their anti-inflammatory and antioxidant properties could have health benefits, including an impact on clinical parameters of the metabolic syndrome that is increasingly present in obese individuals (6). Fat tissue itself is a metabolically active tissue that stimulates the release of pro-inflammatory factors such as TNF-α and interleukin 6 that leads to oxidative stress and creates the preconditions for the development of obesity-related diseases such as cardiovascular disease or diabetes (7).  U posljednjih nekoliko desetljeća zabilježen je porast broja pretilih osoba do razmjera globalne epidemije kao posljedica neadekvatne prehrane, sjedilačkog načina života, ali i genetskih predispozicija. Svjetska zdravstvena organizacija (SZO) proglasila je debljinu najvećim, globalnim, kroničnim, zdravstvenim problemom u odraslih koji se sve više pretvara u ozbiljniji problem od pothranjenosti (1). S druge strane debljina je čimbenik rizika za razvoj kroničnih nezaraznih bolesti današnjice koje čine više od 80% mortaliteta u razvijenim zemljama, poput bolesti krvožilnog sustava, šećerne bolesti, kao i bolesti dišnog sustava poput astme.  Navedeni trend zabilježen je i u Hrvatskoj, kako u odrasloj populaciji tako i kod djece ako se uspoređuju podaci iz 2003. i 2015. godine (2), dok je prema podacima iz 2019. gotovo dvije trećine odraslog stanovništva u Hrvatskoj imalo prekomjernu tjelesnu masu ili bilo pretilo (42% prekomjerna tjelesna masa, 23% pretilost) (3).  Posebice su zabrinjavajući podaci Europske inicijative praćenja debljine iz 2018/2019. koji pokazuju da 35,0% djece dobi od 8,0 do 8,9 godina u RH ima prekomjernu tjelesnu masu i debljinu. Na nacionalnoj razini je problem debljine veći u dječaka nego u djevojčica te iznosi 17,8% u odnosu na 11,9% djevojčica. RH se nalazi na visokom petom mjestu zajedno s ostalim zemljama mediteranske regije Europe zbog odmaka od mediteranskog prema zapadnjačkom načinu prehrane (4).  Suvremeni nutricionistički pristup smanjenju prekomjerne i održavanju normalne tjelesne mase temelji se na promjenama u načinu života, prehrani s energetskim deficitom, redovitoj tjelesnoj aktivnosti te redovitoj kontroli tjelesne mase i fizičke aktivnosti.  Belupo je u suradnji s Podravkom razvio standardnu i inovativnu liniju proizvoda. Radi se o proizvodima koji zamjenjuju jedan ili više dnevnih obroka, a koji moraju biti sukladni Uredbi 2016/1413 koja propisuje zahtjeve vezane uz energetsku vrijednost, sadržaj i sastav masti i bjelančevina te količine vitamina i minerala u navedenim proizvodima (5).  Standardna linija proizvoda sadrži uobičajene sastojke i ova vrsta proizvoda već postoji na svjetskim tržištima dok se inovativna linija proizvoda od standardne razlikuje implementacijom sirovina koje se smatraju boljim izvorima potencijalno bioaktivnih komponenata. Kliničke studije ukazuju da bi navedeni sastojci, zbog svojih protuupalnih i antioksidativnih svojstava mogli imati zdravstvene benefite, između ostalog utjecaj na kliničke pokazatelje metaboličkog sindroma koji je sve češće prisutan kod pretilih osoba (6). Masno tkivo samo po sebi je metabolički aktivno tkivo koje potiče otpuštanje proupalnih čimbenika poput TNF-α i interleukina 6 koji dovode do oksidativnog stresa, i stvaranja preduvjeta za razvoj bolesti povezanih s pretilošću poput kardiovaskularnih bolesti ili dijabetesa (7). |
| --- | --- |
| **Primary outcomes**  **Primarni ciljevi** | To determine the effectiveness of a weight reduction diet program based on an innovative and standard product line through actual reduction of the body mass (BM) / fat mass (FM) of the participants in relation to their pre-trial body mass(BM)/fat mass (FM).  Utvrditi učinkovitost programa prehrane za redukciju tjelesne mase baziranog na inovativnoj i standardnoj liniji proizvoda u stvarnom smanjenju tjelesne mase (TM)/mase masnog tkiva (MMT) ispitanika u odnosu na njihovu tjelesnu masu(TM)/masu masnog tkiva (MMT) prije početka ispitivanja. |
| **Secondary outcomes**  **Sekundarni ciljevi** | 1. reduction of waistline and reduction of waist/hip and waist/height ratio  2. improvement of the lipid profile (increase in HDL/LDL ratio) compared to initial values  3. improvement of the quality of life  4. improvement of asthma control, decrease in the number and severity of asthma exacerbations  5. decrease in systemic inflammation, decrease in oxidant stress biomarkers  1. smanjenje opsega struka i smanjenje omjera struk/bokovi i struk/visina  2. poboljšanje lipidograma (povećanje omjera HDL/LDL) u odnosu na početne vrijednosti  3. poboljšanje kvalitete života  4. Unaprijeđenje kontrole astme, smanjenje učestalosti i težine akutnih egzacerbacija astme  5. smanjenje sistemske upale, smanjenje razine oksidativng stresa |
| **Observational goal**  **Opservacijski cilj** | Improvement and adoption of proper dietary habits  Poboljšanje i usvajanje pravilnih prehrambenih navika |
| **Primary measurements**  **Primarna mjerenja** | Anthropometric measurement once a month:  a. measuring of the body mass and height on a standard scale and a standard altimeter under standard conditions  b. body composition assesment using 2 methods:   1. bioelectrical impedance analysis (BIA) 2. 7-site skinfold measurement – every 2 months   Antropometrijska mjerenja jednom mjesečno:  a. mjerenje tjelesne mase i visine na standardnoj vagi i standardnom visinomjeru u standardnim uvjetima  b. mjerenje sastava tijela korištenjem 2 metode:  1) analiza bioelektričnom impedancom (BIA)  2) mjerenje kožnih nabora (7 mjesta)- svaka 2 mjeseca |
| **Other measurements**  **Ostala mjerenja** | 1. Anthropometric measurements (once a month):   1. waist and hip circumference 2. waist/hip ratio 3. waist/height ratio   2. General physical examination by a doctor (every 2 months)  3. Blood count, biochemistry and inflammation markers (at the beginning of the study and after 3, 6 and 9 months)  a. Lipid profile (triglycerides, total cholesterol, HDL, LDL)  b. Metabolites and substrates (glucose, creatinine, urates, total bilirubin)  c. Liver function test (AST, ALT, GGT, ALP)  d. Inflammatory markers (hs CRP, other adipose tisssue reated biomarkers)  e. Electrolytes (potassium, sodium)  f. Urin analyses, ketone bodies  4. Metagenomic analysis of the stool microbiome (at the begining and the end of the study)  5. Epigenetic and transcriptomic analysis for participans who are part of the same family (at the begining and the end of the study)  6. Spiroergometry (at the beginning and end of the study)  7. Quality of life questionnaire (at the beginning and end of the study)  8. Dietary questionnaires (at the beginning of study ,e and end of the study):   1. Food frequency questionnaire (FFQ) 2. MEDAS (or children version – KIDMED)- 14-point Mediterranean Diet Adherence Screener 3. TFEQ-R1- The Three-Factor Eating Questionnaire   9. Food diary (at the beginning of the study and once in each phase of the study)   1. Antropometrijska mjerenja (jednom mjesečno):    1. opseg struka i bokova    2. omjer struk/bokovi    3. omjer struk/visina. 2. Opći fizikalni pregled liječnika (svaka 2 mjeseca) 3. Laboratorijski parametri (na početku studije te nakon 3, 6 i 9 mjeseci)    1. Lipidogram (trigliceridi, ukupni kolesterol, HDL, LDL)    2. Metaboliti i supstrati (glukoza, kreatinin, urati, ukupni bilirubin)    3. Jetreni enzimi (AST, ALT, GGT, ALP)    4. Upalni čimbenici (hs CRP, drugi biomarkeri vezani uz masno tkivo)    5. Elektroliti (kalij, natrij)    6. Analiza urina, ketonska tijela 4. Metagenomska analiza mikrobioma u stolici (na početku i na kraju studije) 5. Epigenetske i transkriptomske analize u ispitanika koji su dijelom iste obitelji (na početku i na kraju studije) 6. Spiroergometrija (početak i kraj studije) 7. Upitnik o kvaliteti života (početak i kraj studije) 8. Upitnici o prehrambenim navikama FFQ (početak i kraj studije):    1. Food frequency questionnaire (FFQ)    2. MEDAS (or children version – KIDMED)- 14-point Mediterranean Diet Adherence Screener    3. TFEQ-R1- The Three-Factor Eating Questionnaire 9. Dnevnik prehrane (početak, na početku svake faze i kraj studije) |
| **Study duration**  **Trajanje studije** | 40 weeks :  • 0-2 weeks: phase of entering the study  • 2-40 weeks: an intervention phase lasting 40 weeks in which participants consume meal replacement products (MRP) according to the diet program for reducing and maintaining the achieved body mass  40 tjedana:   - 0-2 tjedna: faza ulaska u studiju - 2-40. tjedan: intervencijska faza u trajanju od 40 tjedana u kojoj ispitanici konzumiraju zamjene za obrok (meal replacement product - MRP) prema programu prehrane za redukciju i održavanje postignute tjelesne mase |
| **Participants**  **Ispitanici** | **Adults: 18-60 years, both sexes**  **Children: 12-18 years old, both sexes**  Participants will be randomly assigned to groups with stratification based on age, gender, BMI and % body fat  Odrasli: 18-60 godina, oba spola  Djeca: 12 – 18 godina, oba spola  Ispitanici će biti raspodijeljeni u grupe slučajnim odabirom uz stratifikaciju s obzirom na dob, spol, i BMI i % tjelesne masti |
| **Participant groups**  **Skupine ispitanika** | **Active group:** participants use the innovative line of products intended for body weight reduction, that represent a meal replacement for weight management  **Active control:** participants use the standard line of products intended for weight reduction that represent a meal replacement for weight management with already proven clinical effectiveness (positive control).  **Control group:** participants receive personalized advice on proper nutrition for a reduced low-calorie diet in which they use common food.  Aktivna skupina: ispitanici koriste inovativnu liniju proizvoda namijenjenu redukciji tjelesne mase koji predstavljaju zamjenski obrok za kontrolu tjelesne mase  Aktivna kontrola: ispitanici koriste standardnu liniju proizvoda namijenjenu redukciji tjelesne mase koji predstavljaju zamjenski obrok za kontrolu tjelesne mase s već dokazanom kliničkom učinkovitošću (pozitivna kontrola)  Kontrolna skupina: ispitanici dobivaju personalizirane savjete o pravilnoj prehrani za redukcijsku dijetu u kojoj koriste uobičajene prehrambene namirnice |
| **Sample size**  **Veličina uzorka** | 240 participants (adults and children) in a ratio of 2:1:1 = active group: active control (positive control) group: control group (negative control)  240 ispitanika (odraslih i djece) u omjeru 2:1:1 = aktivna skupina: aktivna kontrola : negativna kontrola |
| **Inclusion criteria**  **Uključuući kriteriji** | • adults aged 18-60, children aged 12-18  • both sexes  • BMI >27 kg/m^2^ (adults); > 90th centile for BMI (children)  • signed informed consent  • odrasli dob od 18-60 godina, djeca dob 12-18 godina  • oba spola  • BMI >27 kg/m2 (odrasli); > 90.centil za BMI (djeca)  • potpisani informirani pristanak |
| **Exclusion criteria**  **Isključujući kriteriji** | - significant comorbidities: uncontrolled diabetes mellitus requiring insulin therapy and other endocrine disorders, cardiovascular disorders and other chronic diseases (including malignancies), chronic inflammatory diseases of the gastrointestinal tract, mental disorder - individuals who are currently on or have recently gone through a weight loss program (in the past 3 months) - individuals who in the past 6 months had an unwanted and uncontrolled loss of body weight >5% - bariatric surgery in the past 6 months - use of medication that increases or decreases appetite - permanent therapy with steroid drugs, antidepressants, cytostatics, hormone therapy, beta-blockers, etc. - eating disorders (bulimia) - individuals with substance abuse issues - pregnancy, breastfeeding - unstable thyroid disease - značajni komorbiditeti: nekontrolirani diabetes mellitus koji zahtjeva inzulinsku terapiju i drugi endokrini poremećaji, kardiovaskularni poremećaji i druge kronične bolesti (uključujući malignitete), kronične upalne bolesti probavnog trakta, psihički poremećaj - osobe koje su trenutno ili su nedavno provodile program mršavljenja (u proteklih 3 mjeseca) - osobe koje su u proteklih 6 mjeseci imale neželjeni i nekontroliran gubitak tjelesne mase >5% - osobe koje su bile podvrgnute barijatrijskoj kirurgiji u proteklih 6 mj. - osobe koje koriste lijekove koji utječu na povećanje ili smanjenje apetita - trajna terapija steroidnim lijekovima, antidepresivima, citostaticima, hormonskom terapijom, beta blokatorima itd. - osobe s poremećajima prehrane (bulimija) - ovisnici o alkoholu i drogama - trudnoća, dojenje - nestabilna bolest štitnjače |
| **Detailed study plan**  **Detaljni plan studije** | The study participantwas designed in such a way that participantin the initial phase participants follow a low-calorie diet of about 1000 - 1100 kcal, whereby 5 daily meals are replaced with meal replacements in the reduction diet (active group and active control) (intensive phase). In subsequent phases, participants gradually increase their energy intake and gradually replace MRP with regular nutritionally profiled meals (active phase and maintenance phase). The final (stable) phase represents the phase in which, in addition to one meal replacement per day, the usual meals designed by the nutritionists of Belupo and the Podravka Culinary Center are consumed, where education and nutrition counseling is also carried out.  Studija je za skupinu odraslih ispitanika koncipirana na način da ispitanici u početnoj fazi provode niskokaloričnu dijetu od oko 1000 - 1100 kcal pri čemu je 5 dnevnih obroka zamijenjeno s zamjenama za obroke kod redukcijske dijete (aktivna skupina i aktivna kontrola) (intenzivna faza). U narednim fazama ispitanici postupno povećavaju energetski unos i postupno zamjenjuju MRP uobičajenim nutritivno profiliranim obrocima (aktivna faza i faza održavanja). Završna (stabilna) faza predstavlja fazu u kojoj se uz jednu zamjenu za obrok dnevno konzumiraju uobičajeni obroci sastavljeni od strane nutricionista Belupa i Kulinarskog centra Podravke, gdje se provodi i edukacija i savjetovanje o prehrani  Pediatric participants in the initial phase of the study implement a low-calorie diet of about 1300-1500 kcal, whereby 3 daily meals are replaced with meal replacements in the reduction diet in addition to 2 nutritionally balanced regular meals that include a minimum of 5 daily servings of fruits and vegetables (active group and active control) (intensive phase).  In the next phase, the participants gradually increase their energy intake and replace one MRP with an additional regular nutritionally profiled meal (active phase). The final (stable) phase represents the phase in which, the usual meals designed by the nutritionists at the Srebrnjak Children`s Hospital are consumed, where education and nutrition counseling is also carried out.  Studija je za dječju populaciju ispitanika koncipirana na način da ispitanici u početnoj fazi provode niskokaloričnu dijetu od oko 1300-1500 kcal pri čemu su 3 dnevna obroka zamijenjena s zamjenama za obroke kod redukcijske dijete uz 2 nutritivno izbalansirana uobičajena obroka koji uključuju minimalno 5 dnevnih serviranja voća i povrća (aktivna skupina i aktivna kontrola) (intenzivna faza).  U narednoj fazi ispitanici postupno povećavaju energetski unos i zamjenjuju jedan MRP dodatnim uobičajenim nutritivno profiliranim obrokom (aktivna faza). Završna (stabilna) faza predstavlja fazu u kojoj se uz jednu zamjenu za obrok dnevno konzumiraju uobičajeni obroci sastavljeni od strane nutricionista DB Srebrnjak gdje se provodi i edukacija i savjetovanje o prehrani.  The usual meals of the later stages of the study, apart from being low in energy and highly nutritionally dense are composed in such a way that they are either ready for consumption as a complete meal (e.g. ready- to eat tuna salad) or can be quickly prepared. In this way, the test participants are directed to an adequate diet, which, by adopting proper food and lifestyle habits, will ultimately enable them to maintain the achieved reduced body weight in the long term.  Uobičajeni obroci kasnijih faza studije, osim što su niske energetske, a visoke nutritivne gustoće, sastavljeni i su na način da su ili već spremni za konzumaciju kao cjelovit obrok (npr. gotova salata od tunjevine) ili se mogu brzo pripremiti. Na taj se način ispitanici upućuju na adekvatnu prehranu koja će im usvajanjem pravilnih prehrambenih i životnih navika naposljetku omogućiti dugoročno održavanje postignute smanjene tjelesne mase.  In addition to MRP, in all phases of the study, the intake of sufficient amounts of fluid (approx. 2 l) in the form of water or unsweetened teas and moderate physical activity (≥150-180 min per week) is encouraged.  The participants will have a choice of consuming savory and sweet MRPs in the form of powdered soups, powdered shakes, liquid shakes, and bars. All products have approximately the same energy value and uniform nutritional composition so that participants can choose the type and flavors of the product according to their own affinities**.**  Uz MRP, u svim fazama studije se potiče unos dovoljnih količina tekućine (cca 2l) u obliku vode ili nezaslađenih čajeva te umjerena tjelesna aktivnost (≥150-180 min na tjedan). Ispitanici će imati na izbor konzumaciju slanih i slatkih MRP-ova u obliku praškastih juha, praškastih shakova, tekućih shakova i pločica. Svi proizvodi imaju približno jednaku energetsku vrijednost i ujednačen nutritivni sastav tako da ispitanici mogu prema vlastitim afinitetima birati vrstu i okuse proizvoda.  **STUDY PHASES**  **FAZE STUDIJE**  **Study entry phases (0-2 weeks):** in this phase, the participants are recruited according to the inclusion and exclusion criteria. Recruitment is carried out by a specialist physician.  During this phase, subjects replace standard meals with meal replacement products and reduce energy intake to a final defined intake depending on the group of subjects.  Faza ulaska u studiju (0-2 tjedna): u ovoj fazi se ispitanici regrutiraju poštujući kriterije uključivanja i isključivanja. Regrutaciju provodi liječnik.  Tijekom ove faze ispitanici zamjenjuju standardne obroke s proizvodima koji predstavljaju zamjenu za obroke i smanjuju energetski unos do konačnog definiranog unosa ovisno o skupini ispitanika .  **ADULT PARTICIPANTS**  The daily energy intake is defined at around 1000-1100 kcal per day, and counseling on nutrition and physical activity is carried out.   1. **Intensive phase adults (2-14 weeks) about 1000 - 1100 kcal/day**   5 MRP + 1 salad + water/unsweetened tea.   1. **Active phase adults (14-21 weeks) 1100-1200 kcal/day**   3 MRP + 2 regular meals (200-280 kcal) + water/unsweetened tea.   1. **Maintenance phase adults (21-28 weeks): 1200-1500 kcal/day**   2 MRP + 3 regular meals (260-350 kcal) + water/unsweetened tea   1. **Stable phase adults (28-40 weeks): 1500-2000 kcal**   1 MRP + 4 regular meals (approx. 320-450 kcal) + water/unsweetened tea  Usual meals can also contain snacks with lower energy values, but it is important to consume approx. 1300-1800 kcal per day with usual foo**d**  **Odrasli ispitanici**  Dnevni energetski unos definiran je na oko 1000 – 1100 kcal na dan te se provodi savjetovanje o prehrani i fizičkoj aktivnosti.  1. Intenzivna faza odrasli (2-14 tjedan oko 1000 - 1100 kcal/dan)  5 MRP + 1 salata + voda/nezaslađeni čaj.  2. Aktivna faza odrasli (14-21 tjedan) 1100-1200 kcal/dan  3 MRP + 2 uobičajena obroka (200-280 kcal) + voda/nezaslađeni čaj.  3. Faza održavanja odrasli (21-28 tjedan),: 1200-1500 kcal/dan  2 MRP + 3 uobičajena obroka (260-350 kcal) + voda/nezaslađeni čaj  4. Stabilna faza odrasli (28-40 tjedan): 1500-2000 kcal  1 MRP + 4 uobičajena obroka (cca 320-450 kcal) + voda/nezaslađeni čaj  Uobičajeni obroci mogu sadržavati i međuobroke nižih energetskih vrijednosti, ali je bitno da se uobičajenim namirnicama unese cca 1300-1800 kcal na dan  **PEDIATRIC PARTICIPANTS**  The daily energy intake is defined at around 1300-1500 kcal per day, and counseling on nutrition and physical activity is carried out.   1. **Intensive phase children (weeks 2-14) around 1300-1500 kcal/day**   3 MPR + 2 regular meals (320-400 kcal) + water + unsweetened tea.   1. **Active phase children (14-21 weeks) around 1400-1600kcal/day**   2 MPR + 3 regular meals (320-400 kcal) + water + unsweetened tea.   1. Maintenance phase (21-28 weeks): 1600-1900 kcal/day 1 MRP + 4 regular meals (350-420 kcal) + water/ unsweetened tea 2. **Stable phase children (week 28-40) 1750-2250 kcal.**   5 regular meals (350-450 kcal) + water/unsweetened tea.  **DJEČJA SKUPINA ISPITANIKA**  Dnevni energetski unos definiran je na oko 1300 – 1500 kcal na dan te se provodi savjetovanje o prehrani i fizičkoj aktivnosti.   1. **Intenzivna faza djeca(2-14 tjedan) oko 1300-1500 kcal/dan**   3 MPR + 2 uobičajena obroka (350-420 kcal) + voda + nezaslađeni čaj.   1. **Aktivna faza djeca (14-21.tjedan) oko 1500-1700kcal/dan**   2 MPR + 3 uobičajena obroka (350-420 kcal) + voda + nezaslađeni čaj.   1. **Stabilna faza djeca (21-28.tjedan) 1700-2000 kcal**   1 MPR + 4 uobicajena obroka (370-450 kcal) + voda/nezasladjeni caj.   1. **Faza praćenja (28-40 tjedan)**   5 uobicajena obroka + voda/nezasladjeni caj.  Snacks are included in regular meals, where the total value of meals and snacks does not exceed the total set value of regular meals.  U običajene obroke su uključeni međuobroci, pri čemu ukupna vrijednost obroka i međuobroka ne prelazi ukupnu zadanu vrijednost uobičajenih obroka.  Participants will have TM measurements and anthropometric measurements once a month, and physician examination every 2 monhts Blood and urine samples for laboratory tests and nutritional status and inflammation level assessments will take place at the beginning of the study, and after 3, 6 and 9 months. Stool samples will be take at baseline and at the end of the study.  At the beginning and at the end of the study, spiroergometry will be performed and questionnaires on quality of life and eating habits are filled out using validated questionnaires.  Smartbands will be used to measure physical activity.  Ispitanici će biti podvrgnuti TM i antropometrijskim mjerenjima jednom mjesečno, dok će fizikalni liječnički pregledi biti svaka 2 mjeseca..  Laboratorijska obrada se za sve ispitanike provodi na početku studije, te nakon nakon 3, 6 i 9 mjeseci. Uzorci stolice prikupljat će se na početku i na kraju studije.  Na početku i na kraju studije provodi se spiroergometrija i ispunjavanje upitnika o kvaliteti života i prehrambenim navikama pomoću validiranih upitnika.  Za mjerenje tjelesne aktivnosti koriste se pametne narukvice za određivanje fizičke aktivnosti.  At the beginning of each phase of the study, all groups of the participants will be consulted about the way to implement the diet in a certain phase and they will be offered customized menus in accordance with their individual needs and the phase of the program they are in.  Counseling on physical activity is also carried out for all participants.  Na početku svake faze studije sve grupe ispitanika savjetovati će se o načinu provođenja prehrane u određenoj fazi i bit će im ponuđeni prilagođeni jelovnici u skladu sa individulanim potrebama i fazi programa u kojoj se nalaze.  Za sve ispitanike provodi se i savjetovanje o fizičkoj aktivnosti |
| **Data analysis**  **Analiza podataka** | Analysis of variance for repeated measurements will be used to determine the effectiveness of individual programs on primary outcomes (BM / FM). The same analysis will be used to analyze secondary performance parameters and safety parameter variables. A chi-square test or Fisher's test will be used to compare the occurrence of safety-related parameters among groups. P<0.05 will be considered statistically significant with appropriate correction for multiple comparisons.  Za utvrđivanje učinkovitosti pojedinih programa na primarne ishode (TM/MMT) koristit će se analiza varijance za ponovljena mjerenja. Ista analiza koristit će se za analizu sekundarnih parametara učinkovitosti te parametrijskih varijabli neškodljivosti. Za usporedbu pojavnosti parametara vezanih uz neškodljivost među skupinama koristit će se hi-kvadrat test ili Fisherov test. P<0,05 smatrat će se statistički značajnim uz odgovarajuću korekciju za višestruke usporedbe. |
| **Risks and benefits of participation**  **Rizici i dobrobiti sudjelovanja** | **Risk:** the products (MRP) are considered safe, and the BM reduction program will be carried out under expert supervision. Possible discomfort may occur during blood sampling (pain at the injection site, bruising).  **Benefit:** personalzed BM reduction program under expert supervision with control of relevant parameters.  All products representing a meal replacement for weight management will be provided by the partners.  Nutritionists from Belupo Nutraceutical Center and Podravka Culinary Center will provide free nutrition counseling and will design personalized menus with reduced energy intake for adult participants. Diet counseling for children will be conducted by nutritionists at the Srebnjak Children's Hospital.  Rizik: proizvod se smatra zdravstveno ispravnim, a program redukcije TM će se vršiti pod stručnim nadzorom. Do eventualne neugodnosti može doći prilikom vađenja krvi (bol na mjestu uboda, podljev).  Dobrobit: besplatan program redukcije TM pod stručnim nadzorom uz kontrolu relevantnih parametara.  Svi proizvodi koji predstavljaju zamjenski obrok za kontrolu tjelesne mase bit će osigurani od strane partnera.  Besplatno savjetovanje o prehrani i izrada personaliziranih jelovnika smanjenog energetskog unosa provest će za odrasle ispitanike nutricionisti Belupo Nutraceutike i Podravka Kulinarskog centra. Proizvodi pod brandom partnera koji će biti navedeni u jelovnicima bit će osigurani od strane partnera Belupo i Podravka. Za dječju populaciju savjetovanje o prehrani provest će nutricionisti DB Srebrnjak. |
| **Study duration**  **Trajanje studije** | 40 weeks  40 tjedana |
| **Vremenski okvir**  **Time frame** | Početak Start__17/01/2023___________  Kraj End _31/12/2023____________  Završno izvješće Final report ___2 years after the end of the study/ 2 gdine nakon završetka provedbe studije_________ |
| **Registration of the study**  **Registracija studije** | The clinical study is registered under www.clinicaltrials.gov. (NCT05980663, NCT05733871)  Registracija studije na www.clinicaltrials.gov. (NCT05980663, NCT05733871) |
| **Ethics related aspects**  **Etički aspekti studije** | This research will respect all national, European, and international laws and ethical rules in research. Data obtained during the research will be fully protected and anonymous except for the attending physician. All patients, samples, and their corresponding clinical data will be assigned a unique study code which will ensure maximum data confidentiality. The research was approved by the Ethics committee (14.03.2022; Reg. no.: 04-301/1-22; Head of Ethics committee: Asst. prof. Prim. Helena Munivrana Škvorc, MD, Ph.D.). All parents/legal guardians and adults participating in the study will have to sign and date the informed consent form approved by the Institutional Ethics Committee before taking part in the study.  Ova studja poštovat će svu relevantnu nacionalnu, europsku i internacionalnu legislativu i etička pravila u istraživanjima. Podaci prikupljeni tijekom studije bit će u potpunosti zaštićeni i anonimizirani, osim za nadležnog liječnika. Svi ispitanici, uzorci i njihovi podaci imat će dodijeljenu jedinstvenu studijsku šifru koja će osigurati maksimalnu zaštitu podataka. Studija je odobrena od strane Etičkog povjerenstva (14.03.2022., klasa i broj: 04-301/1-22, predsjednik Etičkog povjerenstva: doc.dr.sc.prim. Helena Munivrana Škvorc, dr.med.). Svi ispitanici i njihovi roditleji/skrbnici morat će potpisati informirani pristanak odobren od strane Etičkog povjerenstva prije početka sudjelovanja u studiji. |

## Literature/LIteratura

1. D. Štimac, S. Klobučar Majanović, M. Baretić, M. Bekavac Bešlin, A. Belančić, Ž. Crnčević Orlić, V. Đorđević, D. Marčinko, D. Miličić, G. Mirošević, S. Musić Milanović, D. Pavičić Baldani, A. Pokrajac Bulian, S. Rački, D. Rahelić, Ž. Reiner, A. Ružić, M. Samaržija. Hrvatske smjernice za liječenje odraslih osoba s debljinom Croatian guidelines for the treatment of adults with obesity • Acta Med Croatica, 76 (2022) 3-18

2. Milanović MS., Bakul D. (2018): Eidemiologija debljine-javnozdravstveni problem Epidemiology of obesity - a public health problem • Edicus 20188; 27 (1): 7-13.

3. Europka zdravstvena anketa u Hrvatskoj 2019 (European Health Interview Surwey- EHIS)

4. Europska inicijativa praćenja debljine u djece, Hrvatska 2018/2019 (childhood Obesity Surveillanve Initiative, Crotia 2018/2019 (CroSOSi)

5. UREDBA KOMISIJE (EU) 2016/1413 оd 24. kolovoza 2016. o izmjeni Uredbe (EU) br. 432/2012 o utvrđivanju popisa dopuštenih zdravstvenih tvrdnji koje se navode na hrani, osim onih koje se odnose na smanjenje rizika od bolesti te na razvoj i zdravlje djece COMMISSION REGULATION (EU) 2016/1413 of 24 August 2016 amending Regulation (EU) No 182/2011 432/2012 establishing a list of permitted health claims made on foods, other than those relating to the reduction of disease risk and to the development and health of children

6.Ezquerra EA, Vázquez JMC, Barrero AA (2008) Obesity, metabolic syndrome, and diabetes: cardiovascular implications and therapy. Rev Esp Cardiol 61: 752‐764

7. Ellulu, M. S., Patimah, I., Khaza'ai, H., Rahmat, A., & Abed, Y. (2017). Obesity and inflammation: the linking mechanism and the complications. Archives of medical science : AMS, 13(4), 851–863. <https://doi.org/10.5114/aoms.2016.58928>
