# Supplementary figures and images for: A multidisciplinary protocol for reducing excessive and maintaining a healthy body weight in the personalized management of chronic diseases in children and adults
Source: PLoS One. 2025 Mar 13;20(3):e0306400. doi: 10.1371/journal.pone.0306400 (PMC11906058; doi:10.1371/journal.pone.0306400)

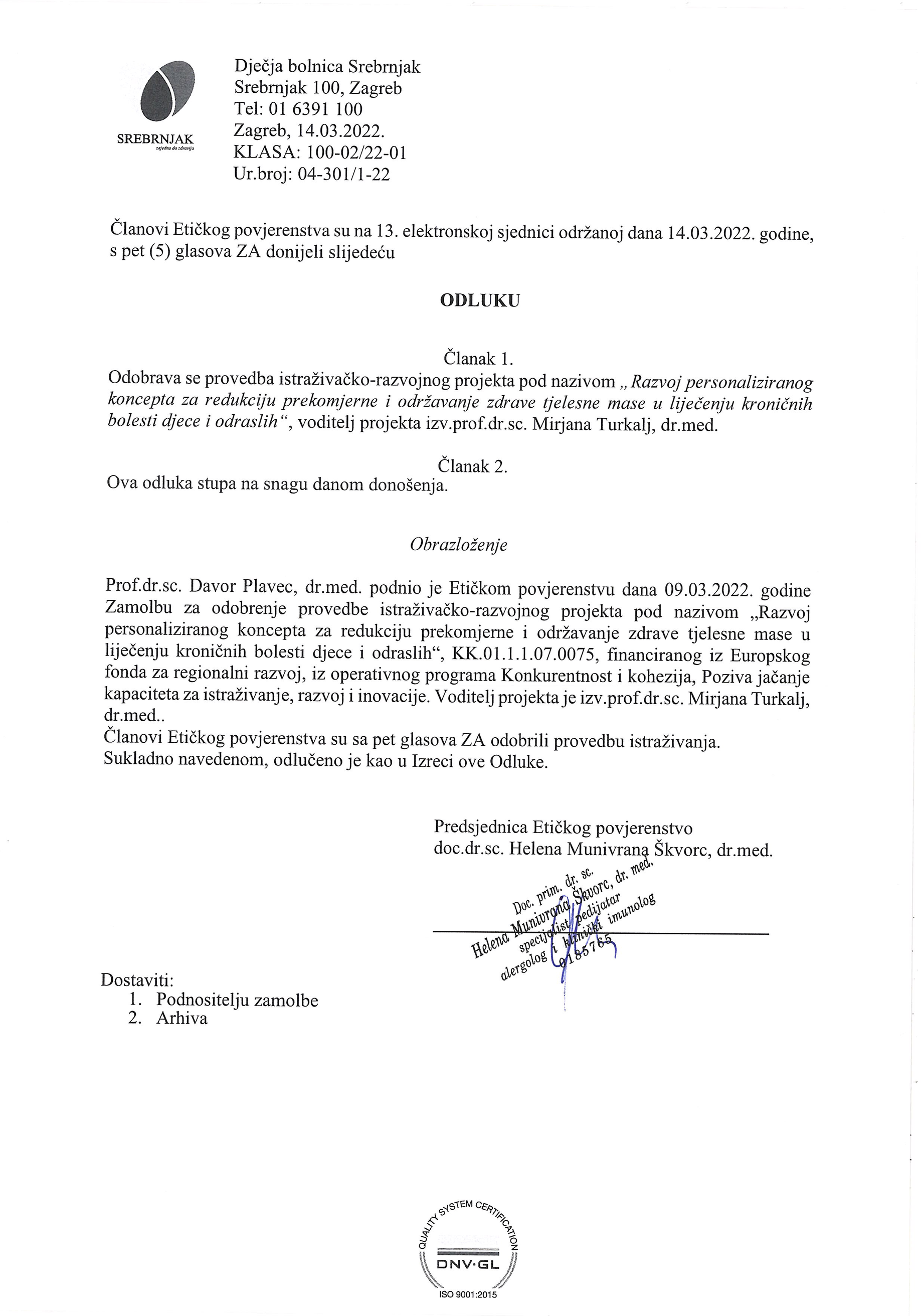

Supplement: S1 Fig — (TIF) [file pone.0306400.s002.tif]
