# Supplementary material for: A multidisciplinary protocol for reducing excessive and maintaining a healthy body weight in the personalized management of chronic diseases in children and adults
Source: PLoS One. 2025 Mar 13;20(3):e0306400. doi: 10.1371/journal.pone.0306400 (PMC11906058; doi:10.1371/journal.pone.0306400)
Supplement: S2 Table — (DOCX) [file pone.0306400.s003.docx]

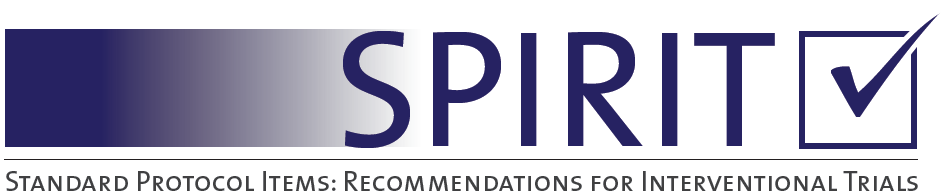


SPIRIT 2013 Checklist: Recommended items to address in a clinical trial protocol and related documents*

| Section/item | ItemNo | Description |
| --- | --- | --- |
| **Administrative information** | | |
| Title | 1 | Randomized stratified controlled clinical study for evaluating the effectiveness of a diet program designed for reducing body weight using standard and innovative products in obese persons with asthma. |
| Trial registration | 2a | Clin trial: NCT05980663, NCT05733871 |
|  | 2b |  |
| Protocol version | 3 | Original, 2023 |
| Funding | 4 | The research is carried out as part of a project funded by the European Regional and Development Fund, Competitiveness and Cohesion OP, Research and Development (KK.01.1.1.07.0075). The funders had no role in study design, data collection and analysis, decision to publish, or preparation of the manuscript. |
| Roles and responsibilities | 5a | Ivana Banić:  Conceptualization  Formal analysis  Investigation  Methodology  Project administration  Writing – original draft  Writing – review & editing  Marija Jankić:  Data curation  Investigation  Writing – original draft  Adrijana Miletić Gospić:  Conceptualization  Data curation  Investigation  Methodology  Writing – original draft  Writing – review & editing  Katarina Pentek:  Conceptualization  Investigation  Methodology  Writing – original draft  Petra Anić:  Investigation  Methodology  Project administration  Writing – original draft  Writing – review & editing  Tajana Burkuš:  Conceptualization  Data curation  Investigation  Methodology  Project administration  Writing – original draft  Krešimir Hrg:  Data curation  Investigation  Methodology  Karmen Zadro:  Investigation  Project administration  Writing – original draft  Jelena Miličević:  Investigation  Methodology  Project administration  Writing – original draft  Ivana Šuljić:  Data curation  Investigation  Methodology  Project administration  Writing – original draft  Marcel Lipej:  Data curation  Investigation  Methodology  Project administration  Writing – original draft  Writing – review & editing  Davor Plavec:  Data curation  Formal analysis  Investigation  Writing – original draft  Lenkica Penava:  Conceptualization  Data curation  Investigation  Methodology  Project administration  Writing – original draft  Writing – review & editing  Mirjana Turkalj:  Conceptualization  Data curation  Investigation  Methodology  Project administration  Supervision  Writing – original draft  Writing – review & editing |
|  | 5b | Jelena Miličević:  Investigation  Methodology  Project administration  Writing – original draft  Ivana Šuljić:  Data curation  Investigation  Methodology  Project administration  Writing – original draft  Marcel Lipej:  Data curation  Investigation  Methodology  Project administration  Writing – original draft  Writing – review & editing  Davor Plavec:  Data curation  Formal analysis  Investigation  Writing – original draft  Lenkica Penava:  Conceptualization  Data curation  Investigation  Methodology  Project administration  Writing – original draft  Writing – review & editing  Mirjana Turkalj:  Conceptualization  Data curation  Investigation  Methodology  Project administration  Supervision  Writing – original draft  Writing – review & editing  Name and contact information for the trial sponsor  This study is funded by the European Regional Development Fund, the Operational Program for Competitiveness and Cohesion, calls for capacity building for research, development and innovation (KK.01.1.1.07.0075). |
|  | 5c | Role of study sponsor and funders, if any, in study design; collection, management, analysis, and interpretation of data; writing of the report; and the decision to submit the report for publication, including whether they will have ultimate authority over any of these activities  This funding source had no role in the design of this study and will not have any role during its execution, analyses, interpretation of the data, or decision to submit results. |
|  | 5d | Composition, roles, and responsibilities of the coordinating centre, steering committee, endpoint adjudication committee, data management team, and other individuals or groups overseeing the trial, if applicable (see Item 21a for data monitoring committee)  NA |
| Introduction |  |  |
| Background and rationale | 6a | Description of research question and justification for undertaking the trial, including summary of relevant studies (published and unpublished) examining benefits and harms for each intervention  Introduction (p. 1-3) |
|  | 6b | Explanation for choice of comparators  Materials and Methods, section Study protocol, p. 9 |
| Objectives | 7 | Specific objectives or hypotheses  Introduction (p. 3) |
| Trial design | 8 | Description of trial design including type of trial (eg, parallel group, crossover, factorial, single group), allocation ratio, and framework (eg, superiority, equivalence, noninferiority, exploratory)  Materials and Methods (p. 7-9)  Supplement 1 (Study protocol) |
| Methods: Participants, interventions, and outcomes | | |
| Study setting | 9 | Description of study settings (eg, community clinic, academic hospital) and list of countries where data will be collected. Reference to where list of study sites can be obtained  Materials and Methods (p. 3-4) |
| Eligibility criteria | 10 | Inclusion and exclusion criteria for participants. If applicable, eligibility criteria for study centres and individuals who will perform the interventions (eg, surgeons, psychotherapists)  Materials and Methods, section Study population (p. 4-6) |
| Interventions | 11a | Interventions for each group with sufficient detail to allow replication, including how and when they will be administered  Materials and Methods, section Study protocol (p. 7-9),  Supplement 1 (Study protocol) |
|  | 11b | Criteria for discontinuing or modifying allocated interventions for a given trial participant (eg, drug dose change in response to harms, participant request, or improving/worsening disease)  Materials and Methods, section Follow up (p. 7-9) |
|  | 11c | Strategies to improve adherence to intervention protocols, and any procedures for monitoring adherence (eg, drug tablet return, laboratory tests)  Materials and Methods, section Follow up (p. 7-9) |
|  | 11d | Relevant concomitant care and interventions that are permitted or prohibited during the trial  Materials and Methods, section Follow up (p. 7-9) |
| Outcomes | 12 | Primary, secondary, and other outcomes, including the specific measurement variable (eg, systolic blood pressure), analysis metric (eg, change from baseline, final value, time to event), method of aggregation (eg, median, proportion), and time point for each outcome. Explanation of the clinical relevance of chosen efficacy and harm outcomes is strongly recommended.  Results, Table 1 (p. 10) |
| Participant timeline | 13 | Time schedule of enrolment, interventions (including any run-ins and washouts), assessments, and visits for participants. A schematic diagram is highly recommended  Materials and Methods, section Follow up (p. 7-9),  Fig 1 |
| Sample size | 14 | Estimated number of participants needed to achieve study objectives and how it was determined, including clinical and statistical assumptions supporting any sample size calculations  Materials and Methods, section Statistical analysis (p. 10) |
| Recruitment | 15 | Strategies for achieving adequate participant enrolment to reach target sample size  Materials and Methods, section Statistical analysis (p. 10), Discussion (p. 14) |
| **Methods: Assignment of interventions (for controlled trials)** | | |
| Allocation: |  |  |
| Sequence generation | 16a | Method of generating the allocation sequence (eg, computer-generated random numbers), and list of any factors for stratification. To reduce predictability of a random sequence, details of any planned restriction (eg, blocking) should be provided in a separate document that is unavailable to those who enrol participants or assign interventions  Materials and Methods, (p. 7-9), Supplement Table 2 |
| Allocation concealment mechanism | 16b | Mechanism of implementing the allocation sequence (eg, central telephone; sequentially numbered, opaque, sealed envelopes), describing any steps to conceal the sequence until interventions are assigned  Materials and Methods (p. 7-9), Supplement Table 2 |
| Implementation | 16c | Who will generate the allocation sequence, who will enrol participants, and who will assign participants to interventions  Materials and Methods (p. 7-9), Supplement Table 2 |
| Blinding (masking) | 17a | Who will be blinded after assignment to interventions (eg, trial participants, care providers, outcome assessors, data analysts), and how  Materials and Methods (p. 7-9), Supplement Table 2 |
|  | 17b | If blinded, circumstances under which unblinding is permissible, and procedure for revealing a participant’s allocated intervention during the trial  Materials and Methods (p. 7-9), Supplement Table 2 |
| **Methods: Data collection, management, and analysis** | | |
| Data collection methods | 18a | Plans for assessment and collection of outcome, baseline, and other trial data, including any related processes to promote data quality (eg, duplicate measurements, training of assessors) and a description of study instruments (eg, questionnaires, laboratory tests) along with their reliability and validity, if known. Reference to where data collection forms can be found, if not in the protocol  Materials and Methods, section Assessments, measurements, diagnostic procedures and data collection (p. 6-7) |
|  | 18b | Plans to promote participant retention and complete follow-up, including list of any outcome data to be collected for participants who discontinue or deviate from intervention protocols  Materials and Methods, section Follow up (p .7-9) |
| Data management | 19 | Plans for data entry, coding, security, and storage, including any related processes to promote data quality (eg, double data entry; range checks for data values). Reference to where details of data management procedures can be found, if not in the protocol  Supplement Fig 1, Informed consent- manuscript p. 6-7 |
| Statistical methods | 20a | Statistical methods for analysing primary and secondary outcomes. Reference to where other details of the statistical analysis plan can be found, if not in the protocol  Materials and Methods, section Statistical analysis (p. 10) |
|  | 20b | Methods for any additional analyses (eg, subgroup and adjusted analyses)  Materials and Methods, section Statistical analysis (p. 10) |
|  | 20c | Definition of analysis population relating to protocol non-adherence (eg, as randomised analysis), and any statistical methods to handle missing data (eg, multiple imputation) |
| **Methods: Monitoring** | | |
| Data monitoring | 21a | Composition of data monitoring committee (DMC); summary of its role and reporting structure; statement of whether it is independent from the sponsor and competing interests; and reference to where further details about its charter can be found, if not in the protocol. Alternatively, an explanation of why a DMC is not needed  Not applicable, this is not a classical clinical study, and the intervention measures are not drugs or medications but food supplements, diet and physical activity. |
|  | 21b | Description of any interim analyses and stopping guidelines, including who will have access to these interim results and make the final decision to terminate the trial NA |
| Harms | 22 | Plans for collecting, assessing, reporting, and managing solicited and spontaneously reported adverse events and other unintended effects of trial interventions or trial conduct  Materials and Methods, section Follow up (p. 7-9) |
| Auditing | 23 | Frequency and procedures for auditing trial conduct, if any, and whether the process will be independent from investigators and the sponsor NA |
| Ethics and dissemination | | |
| Research ethics approval | 24 | Plans for seeking research ethics committee/institutional review board (REC/IRB) approval  Materials and Methods, section Study population (p. 4)  Supplement 1 (Ethic approval) |
| Protocol amendments | 25 | Plans for communicating important protocol modifications (eg, changes to eligibility criteria, outcomes, analyses) to relevant parties (eg, investigators, REC/IRBs, trial participants, trial registries, journals, regulators) NA |
| Consent or assent | 26a | Who will obtain informed consent or assent from potential trial participants or authorised surrogates, and how (see Item 32)  Materials and Methods, section Study population (p. 4)  Supplement, manuscript p. 5-7 |
|  | 26b | Additional consent provisions for collection and use of participant data and biological specimens in ancillary studies, if applicable  Supplement, (informed consent)- manuscript p. 5-7 |
| Confidentiality | 27 | How personal information about potential and enrolled participants will be collected, shared, and maintained in order to protect confidentiality before, during, and after the trial  Supplement, (informed consent) p. 5-7 |
| Declaration of interests | 28 | Financial and other competing interests for principal investigators for the overall trial and each study site NA |
| Access to data | 29 | Statement of who will have access to the final trial dataset, and disclosure of contractual agreements that limit such access for investigators  Supplement, (informed consent) p. 5-7 |
| Ancillary and post-trial care | 30 | Provisions, if any, for ancillary and post-trial care, and for compensation to those who suffer harm from trial participation  Discussion (p. 13) |
| Dissemination policy | 31a | Plans for investigators and sponsor to communicate trial results to participants, healthcare professionals, the public, and other relevant groups (eg, via publication, reporting in results databases, or other data sharing arrangements), including any publication restrictions  Results (p. 11-12), Discussion 13)  Supplement Figure 1, informed consent- manuscript p. 5-7 |
|  | 31b | Authorship eligibility guidelines and any intended use of professional writers NA |
|  | 31c | Plans, if any, for granting public access to the full protocol, participant-level dataset, and statistical code NA |
| Appendices |  |  |
| Informed consent materials | 32 | Model consent form and other related documentation given to participants and authorised surrogates  Supplement Figure 1, Informed consent- manuscript p.5-7 |
| Biological specimens | 33 | Plans for collection, laboratory evaluation, and storage of biological specimens for genetic or molecular analysis in the current trial and for future use in ancillary studies, if applicable  Supplement Figure 1, Informed consent- manuscript p. 5-7 |

*It is strongly recommended that this checklist be read in conjunction with the SPIRIT 2013 Explanation & Elaboration for important clarification on the items. Amendments to the protocol should be tracked and dated. The SPIRIT checklist is copyrighted by the SPIRIT Group under the Creative Commons “[Attribution-NonCommercial-NoDerivs 3.0 Unported](http://www.creativecommons.org/licenses/by-nc-nd/3.0/)” license.

Supplement 1- ethic approval

Supplement 2- study protocol
